# Manganism – Unusual Presentation at Memory Assessment Service

**DOI:** 10.1192/bjo.2025.10760

**Published:** 2025-06-20

**Authors:** Thilini Ranasinghe

**Affiliations:** Kent and Medway Social Care Partnership Trust, Kent, United Kingdom

## Abstract

**Aims:** More than 200,000 clients are referred to memory assessment annually in the United Kingdom. Alzheimer’s disease and vascular brain injury are found to be the main causes for the memory impairment among these clients. However, a minority of clients present with memory impairment due to metabolic causes.

**Methods:** Mr M, a 65-year-old Caucasian male was referred to memory assessment service due to memory problems for 7 months duration. He had evidence of amnesia, aphasia and apraxia. His executive functions, recognition, personality were intact. He scored 91/100 in Addenbrooke’s cognitive examination. M also struggled with balance and tremors of his limbs.

He was diagnosed with liver impairment secondary to metabolic syndrome, type II diabetes, hypertension, long-standing cervical pain and heart block. He reported to sleep more than usual and was suffering from frequent episodes of constipation which was exacerbated by morphine. His partner reported that his cognitive symptoms coincides with constipation.

M was on treatment for mixed anxiety and depressive disorder with sertraline for 4 years. He was euthymic at presentation.

His laboratory work showed mild anaemia and low platelets. He was known to have a platelet disorder as well. Most recent HbA1c was raised but other basic blood investigations were largely within normal ranges.

His magnetic resonance imaging scan showed Symmetrical T1 high signal in bilateral globus pallidus on sagittal T1 weighted images. It was concluded that appearances could be due to manganese deposition consistent with history of hepatic dysfunction.

Small vessel ischaemic changes were seen in bilateral supratentorial white matter.

His electro encephalogram was in keeping with diffuse cerebral dysfunction.

Neurology multi-disciplinary meeting has concluded that the clinical presentation is one of a hepatic encephalopathy.

**Results:** Human physiological functions require many essential elements and manganese is identified as an essential element. Accumulation of manganese in excessive amounts in brain due to various metabolic derangements can causes central nervous system dysfunction known as Manganism. Manganism is an extrapyramidal disorder characterized by motor disturbances associated with neuropsychiatric and cognitive disabilities similar to Parkinsonism.

Manganese is cleared from the body by the liver. Chronic liver impairment hinders the clearing process causing accumulation of manganese in blood and brain. M was suffering from chronic liver impairment which was the most likely cause for manganese deposition in his brain.

**Conclusion:** It was concluded that M’s cognitive impairment was due to hepatic encephalopathy and Manganism. Clinicians need to be aware of Manganism while assessing the patients with chronic liver impairment and neurocognitive dysfunction.

